# Efficacy and safety of approved cellular therapies and bispecific antibodies in solid tumors: the current state

**DOI:** 10.1007/s12672-026-04802-x

**Published:** 2026-03-17

**Authors:** Rabab Zehra Jafry, Saba Bilal Qamar, Sohaib Irfan, Muhammad Abbas Abid, Virginia Mohlere, Neha Maithel, Syed Hassan Jafri, Sanjay Awasthi, Samer Srour, Irfan A. Vaziri, Muhammad Bilal Abid

**Affiliations:** 1 The Aga Khan University School of Medicine, Karachi, Pakistan; 2 Department of Hematology/Oncology, The University of Texas Health Science Center at Houston, Houston, TX USA; 3 Department of Stem Cell Transplantation and Cellular Therapy, The University of Texas MD Anderson Cancer Center, Houston, TX USA; 4 Cancer Partners of Nebraska, Lincoln, NE USA; 5 Division of Hematology/Oncology, Department of Internal Medicine, McGovern Medical School, UTHealth, 6410 Fannin St. | Suite 722 |, 77030 Houston, TX USA

**Keywords:** Bispecific antibodies, T-cell receptor therapy, Tumor-infiltrating lymphocytes, Efficacy, Safety, Lung cancer, Metastatic melanoma, Synovial sarcoma

## Abstract

Cancer immunotherapy is a broad term including naked antibodies, bispecific antibodies (BsAbs), immune-checkpoint inhibitors (ICIs), chimeric antigen receptor T-cell (CAR-T) therapy, cancer vaccines, activated immune cells, and allogeneic hematopoietic cell transplantation. Recent advances in immune-engaging T-cell therapies have led to significant progress in novel treatments across various cancer types, resulting in prolonged patient survival, mainly in patients with hematologic malignancies. Several CAR T-cell constructs and BsAbs have been approved by regulatory authorities worldwide for relapsed, refractory leukemia, non-Hodgkin lymphoma, and multiple myeloma. Solid tumors are characterized by an immunosuppressive, fibroinflammatory tumor microenvironment (TME) and immune-suppressive cells, such as regulatory T-cells (Tregs), myeloid-derived suppressor cells (MDSCs), and tumor-associated macrophages (TAMs). This, coupled with challenges such as tumor-intrinsic factors (tumor antigen heterogeneity, target antigen loss, hypoxia within the tumor bed, and insufficient preexisting tumor-infiltrating lymphocytes [TILs]) and therapy-related hurdles like on-target off-tumor toxicity, impedes the development of durable immune-engaging T-cell therapies across solid tumor types. However, a few BsAbs, TILs, and autologous-engineered T-cell receptor (TCR) therapies have recently been approved across indications, paving the way for newer horizons in the solid tumor cellular therapy landscape. Here, we review the available data on the efficacy and safety of cellular therapies and BsAbs in solid tumors approved thus far by the U.S. Food and Drug Administration. These include BsAbs, TILs, and TCR therapy. Given the lack of homogeneous clinical practice guidelines for TCE therapies in solid tumors, we discuss and compare the efficacy and safety of five therapies. We further present the therapeutic pipeline across solid tumors and discuss strategies currently under investigation to optimize T-cell subsets to enhance antitumor efficacy while improving the safety profile.

## Introduction

Recent advances in immune-engaging T-cell therapies have led to significant progress in novel treatments across various cancer types, resulting in prolonged patient survival. Several T-cell–engaging (TCE) platforms, primarily chimeric antigen receptor (CAR) T-cell products and bispecific antibodies (BsAbs), have been approved by regulatory authorities worldwide for hematologic malignancies [1–3]. By contrast, solid tumors are difficult to treat and have not had any TCEs approved until very recently. This is primarily due to an immunosuppressive tumor microenvironment (TME), which includes cells like regulatory T-cells (Tregs), myeloid-derived suppressor cells (MDSCs), and tumor-associated macrophages (TAMs), and other challenges such as tumor antigen heterogeneity, hypoxia, and insufficient pre-existing tumor-infiltrating lymphocytes (TILs), alongside therapy-related hurdles like on-target off-tumor toxicity [4]. The therapeutic goals of T-cell therapies include navigating the complex TME of solid tumors, enhancing tumor specificity, minimizing off-target toxicity, and improving patient outcomes across tumor types.

In our recent work, we summarized the approved BsAbs for hematologic malignancies [6]. This review discusses efficacy and safety data related to cellular therapies and BsAbs approved thus far in solid tumors. These include BsAbs, TILs, and autologous-engineered T-cell receptor (TCR) therapy. Given the lack of homogeneous clinical practice guidelines for TCE therapies in solid tumors, we further discuss and compare the efficacy and safety of five therapies approved by the U.S. Food and Drug Administration (FDA): amivantamab, tarlatamab, lifileucel, afamitresgene autoleucel, and tebentafusp-tebn, to lay the foundation for future treatment guidelines (Table 1).

**Table 1:** Efficacy and toxicity data for approved T-cell engaging immunotherapies in solid tumors

	Amivantamab	Tarlatamab	Lifileucel	Tebentafusp-tebn	Afamitresgene autoleucel
Clinical trial	MARIPOSA-2	DeLLphi-301	C-144-01	IMCgp100-202	SPEARHEAD-1
FDA-approved indication	NSCLC (exon 20)	ES-SCLC	Unresectable/ metastaticmelanoma	Unresectable/ metastatic uveal melanoma	Synovial sarcoma
Approval date	8/19/2024	5/16/2024	2/16/2024	1/25/2022	8/2/2024
Type of cellular therapy	BsAb	BsAb	TIL	BsAb	Autologous TCR
Target antigen	Extracellular domains of EGFR and MET	DLL3 × CD3	Endogenous tumor antigens	GP100 × CD3	MAGE-A4
Dose & dosing schedule	1400 mg q1w for 4w; 1750 mg q3w thereafter*	10 mg q2w	7.5 × 10^9^–72 × 10^9^ viable cells	20 µg on d1, 30 µg on d8, 68 µg weekly thereafter**	2.68 × 10^9^–10 × 10^9^ cells***
ORR	AC: 64% ALC: 63% Chemo: 36%	10 mg: 40% 100 mg: 32%	31.5%§	T: 9% C: 5%	43.2%
CR	NR	NR	8	NR	2
mPFS, months (95% CI)	AC: 6.3 (5.6–8.4) ALC: 8.3 (6.8–9.1) Chemo: 4.2 (4.0–4.4.0.4)	10 mg: 4.9 (2.9–6.7) 100 mg: 3.9 (2.6–4.4)	4.1 (2.8–4.4)	T: 3.3 (3–5) C: 2.9 (2.8–3.8)	3.8 (2.8–5.8)
mDOR,months	AC: 6.9 ALC: 9.4 Chemo: 5.6	Not evaluable	Not reached	T: 9.9 C: 9.7	6
CRS					
Overall	NR	10 mg: 68 (51%) 100 mg: 53 (61%)	NR	T: 217 (89%) C: 3 (3%)	37 (71%)
Grade ≥ 3	NR	10 mg: 1 (1%) 100 mg: 5 (6%)	NR	T:2 (1%) C: 0 (0%)	1(1.4%)
Median (range) time of onset	NR	13.1 (7.8–27.4) hr	NR	Within 24 h	2.5 (1–5) d
Duration (range)	NR	4 (2–6) d	NR	Few hr–3 d	3 (1–14) d
Treatment	NR	Supportive care†	NR	Antipyretic agents, IV fluids, glucocorticoids, or combination-	Tocilizumab‡
ICANS					
Overall	NR	10 mg: 11 (8%) 100 mg: 24 (28%)	NR	NR	NR
Grade ≥ 3	NR	10 mg: 0 100 mg: 4 (5%)	NR	NR	NR
Neutropenia					
Overall	AC: 74 (57%) ALC: 181 (69%) Chemo: 101 (42%)	10 mg: 23 (17%) 100 mg: 14 (16%)	37 (56%)	NR	NR
Grade ≥ 3	AC: 59 (45%) ALC: 144 (55%) Chemo: 52 (21%)	10 mg: 8 100 mg: 9	26 (39%)	NR	44 (85%)
Infection					
Overall	AC: 27 (21%) ALC: 44 (17%) Chemo: 25 (10%)	41%	NR	NR	NR
Grade ≥ 3	AC: 2 (2%) ALC: 0 Chemo: 0	13%	NR	NR	NR
Infusion-related reactions	AC: 76 (58%) ALC: 148 (56%) Chemo: 1 (0.4%)	NR	6 (3.8%)	NR	NR

BsAb, bispecific antibodies; ES-SCLC, extensive-stage small cell lung cancer; IV, intravenous; NR, not reported; NSCLC, non–small cell lung cancer; TCR, T-cell receptor; TIL, tumor-infiltrating lymphocytes; AC, amivantamab-chemotherapy; ALC, amivantamab-lazertinib-chemotherapy; Chemo, chemotherapy only; CR, complete response; CRS, cytokine release syndrome; ICANS, Immune effector cell–associated neurotoxicity syndrome; mDOR, median duration of response; mPFS, median progression-free survival; NR, not reported; ORR, overall response rate; T, tebentafusp arm; C, control group

* (Combination therapy dosing): For patients weighing ≥ 80 kg, dosages were 1750 mg weekly for the first 4 weeks and 2100 mg every 3 weeks thereafter. The first infusion was split over 2 days, with 350 mg on cycle 1, day 1 and the remainder on cycle 1, day 2. Lazertinib was administered orally at 240 mg daily

** Pembrolizumab was administered IV at a dose of 2 mg/kg; ipilimumab was administered IV at a dose of 3 mg/kg on day 1 of each 21-day cycle for a maximum of 4 doses. Dacarbazine was administered IV at a dose of 1000 mg/m^2^ of body-surface area

*** Administered in one or more infusion bags

§ 36% in 66 patients who progressed after immunotherapy/targeted therapy

† Acetaminophen, intravenous hydration, and glucocorticoids, alone or in combination

‡ For grade 1 CRS lasting ≥ 24 h or for grade 2 CRS

# Median time of onset, 5 days

### Amivantamab

Amivantamab is a fully humanized bispecific antibody that binds to the extracellular domains of EGFR (Kd 1.4nM/L) with no agonist activity and prevents hepatocyte growth factor from engaging the MET (Kd 40picoM/L) receptor. Due to its low fucose content, it can augment antibody-dependent cellular cytotoxicity through activated immune cells. This may contribute to its distinctive safety profile (includes a high rate of infusion-related reactions). There is a preferential blockade of MET over EGFR. It has been approved for the treatment of advanced non–small cell lung cancer (NSCLC) with an EGFR exon 20 insertion mutation. While this BsAb does not engage endogenous T-cells, amivantamab has three main functions: blockade of ligands, degradation of receptors, and stimulation of immune cells [7–9]. BsAb constructs enable the immune system to selectively target tumor cells. They are designed to bind concurrently to two distinct epitopes. These epitopes can be located on different antigens or on separate sites of the same antigen. Typically, one end of the BsAb binds to the host’s CD3 receptor on the T-cells, while the other binds to a specific antigen/epitope on the tumor cell surface, leading to activation of the T-cells and immune-mediated cell death [10].

On May 21, 2021, the FDA granted accelerated approval for amivantamab (amivantamab-vmjw) for locally advanced or metastatic NSCLC based on the results of the phase-I CHRYSALIS trial. In this trial, amivantamab was administered to patients with EGFR exon 20 insertion mutations. Amivantamab was given intravenously at 1,050 mg for patients weighing < 80 kg and 1,400 mg for those weighing ≥ 80 kg. The BsAb was administered weekly for the first 4 weeks, then every 2 weeks from week 5 onwards [11]. While the overall response rate (ORR) was 40%, there were 3 complete responses (CR). The median duration of response (mDOR) was 11.1 months, with a median progression-free survival (PFS) of 8.3 months. Adverse events (AEs) included rash (86%), infusion-related reactions (IRR, 66%), paronychia (45%), hypoalbuminemia (27%), stomatitis (21%), peripheral edema (18%), pruritus (17%), diarrhea (12%), interstitial lung disease (4%), and neutropenia (4%). 30% of patients experienced serious AEs (*n* = 34/114). Pulmonary embolism and back pain are 3% each, being the most common among the serious AEs. One case each of infected dermal cysts, atrial flutter, pneumonitis, cellulitis, and toxic epidermal necrolysis was also observed. 4% (4%) of patients discontinued the trial due to AEs, primarily due to IRR [12, 13].

On March 1, 2024, the FDA approved amivantamab in combination with chemotherapy (carboplatin and pemetrexed) for first-line treatment of locally advanced or metastatic NSCLC with EGFR exon 20 insertion mutations, based on the results from the phase-III PAPILLON trial [14]. In this trial, treatment-naïve patients with advanced NSCLC were given intravenous amivantamab plus chemotherapy (amivantamab arm) or chemotherapy alone (carboplatin and pemetrexed). The median treatment duration was 9.7 months in the amivantamab arm and 6.7 months in the chemotherapy arm. With a median follow-up of 14.9 months, the ORR and mDOR were 73% and 9.7 months in the amivantamab arm, compared to 47% and 4.4 months in the chemotherapy arm, respectively. The mPFS was 11.4 months in the amivantamab arm, compared to 6.7 months in the chemotherapy arm. IRR was reported in 42% of the amivantamab–chemotherapy group and 1% in the chemotherapy group [15]. Neutropenia was reported in 59% of the amivantamab recipients and 45% of the chemotherapy group. COVID-19 occurred in 24% of the amivantamab arm and 14% in the chemotherapy arm [16].

On August 19, 2024, based on the results from the phase-III MARIPOSA trial, the FDA approved amivantamab in combination with lazertinib for the first-line treatment of adult patients with locally advanced or metastatic NSCLC harboring EGFR exon 19 deletions or exon 21 L858R substitution mutations [17]. The FDA then approved amivantamab with chemotherapy (carboplatin and pemetrexed) for refractory EGFR-mutated NSCLC (with EGFR exon 19 deletions or exon 21 L858R substitution mutations) on September 19, 2024, based on the phase-III MARIPOSA-2 trial [18]. The 3 study arms included amivantamab–lazertinib–chemotherapy, amivantamab–chemotherapy, and chemotherapy, with respective PFS of 8.3, 6.3, and 4.2 months for each group. The ORR was 63%, 64%, and 36% across the 3 groups, and the mDOR was 9.4, 6.9, and 5.6 months, respectively. Grade 3 AEs included thrombocytopenia, neutropenia, leukopenia, and anemia. Neutropenia occurred in 69%, 57%, and 42% of patients in the amivantamab–lazertinib–chemotherapy, amivantamab–chemotherapy, and chemotherapy groups, respectively. Of these, grade 3 neutropenia occurred in 55%, 45%, and 21% of patients, respectively. IRRs were reported in 56%, 58%, and 0.4% of patients across the 3 groups, respectively. COVID-19 was reported in 21%, 17%, and 10% of patients across the 3 groups, with grade 3 infections occurring in 2% of the amivantamab–chemotherapy group and none in the other two groups [19]. Overall, the most significant finding in this trial is the improvement in PFS in the study arms that included amivantamab. Additionally, the mDOR and ORR were higher in the regimens that contained amivantamab.

The updated overall survival (OS) data from the MARIPOSA study were presented at the European Lung Cancer Congress (ELCC) 2025 and confirmed improved efficacy for amivantamab–lazertinib combination over Osimertinib in first line for EGFR-mutated NSCLC. The hazard ratio for the OS was 0.75, hence conferring a 25% survival benefit with the combination of amivantamab–lazertinib. Additionally, 60% of patients who received the combination were alive at 3 years compared to 51% of those who received Osimertinib, with continued benefit shown at 42 months (56% vs. 44%, respectively). While the median OS for the Osimertinib arm was 36 months, patients who received amivantamab–lazertinib had not reached median OS yet. This established the combination as the new standard of care over Osimertinib alone, ^20,21^.

Based on the safety profile across all registrational trials, amivantamab can cause major AEs, including rash, IRR, fatigue, nail changes, nausea, constipation, edema, stomatitis, decreased appetite, and musculoskeletal pain [22]. To mitigate IRRs, patients are premedicated with dexamethasone, diphenhydramine, and acetaminophen. Importantly, administration of dexamethasone 8 mg on days − 2, −1, and immediately before infusion markedly reduced IRR occurrence. There was a further reduction in the incidence of IRR observed with the subcutaneous route compared to intravenous [23]. Studies of underlying mechanisms suggest that IRRs are not associated with complement activation, mast cell degranulation, cytokine release syndrome, and tumor lysis syndrome. Unlike the other BsAbs discussed in this review, no cytokine release syndrome (CRS) was reported in any of the three clinical trials. This is due to the unconventional bispecific construct not engaging endogenous T-cells. Several studies are currently underway to address the safety issues associated with amivantamab. Particularly, the results from phase-III, international, randomized PALOMA-3 trial demonstrated non-inferiority of subcutaneous amivantamab compared to IV formulation in terms of efficacy and safety. Similarly, the SKIPirr study specifically focused on the reduction of IRRs associated with subcutaneous administration of amivantamab. Additionally, the open-label, phase-II COCOON study investigated the effectiveness of an enhanced preventive regimen and reducing moderate-to-severe dermatologic AEs in patients with the EGFR-mutated NSCLC. Initial results demonstrate that the COCOON regimen significantly reduced the incidence of moderate-to-severe dermatologic reactions [23, 24].

### Tarlatamab

Tarlatamab, a BsAb targeting DLL3 and CD3, is FDA-approved for the treatment of extensive-stage small cell lung cancer (ES-SCLC) following disease progression on or after platinum-based chemotherapy. In the DeLLphi-301 phase-II clinical trial, tarlatamab was administered intravenously to patients in two doses every 2 weeks: 10 mg (*n* = 100) and 100 mg (*n* = 88). Patients received intravenous 8 mg dexamethasone premedication before tarlatamab infusion on day 1 and day 8 to mitigate CRS risk. Other measures included prophylactic hydration and a step dosing approach. The ORR was 40% in the 10 mg cohort and 32% in the 100 mg cohort. The mPFS was 4.9 months and 3.9 months in the respective dose cohorts. The DeLLphi-301 trial concluded that 10 mg dosing had similar efficacy and a better safety profile than the 100 mg dose.

In terms of the safety profile, 30% (*n* = 40/133) of patients in the 10 mg arm and 32% (*n* = 28/87) in the 100 mg group experienced grade 1 CRS, whereas grade 2 CRS occurred in 20% (*n* = 27/133) and 23% (*n* = 20/87) of patients, respectively. Most cases occurred after the first two doses. Grade 3 CRS was observed in 1% of patients in the 10 mg group and 6% of patients in the 100 mg group. Most cases of CRS were managed with supportive care that included glucocorticoids. Additional interventions were seldom required and included tocilizumab (in 5% of patients in the 10-mg group and 10% in the 100-mg group), supplemental oxygen (in 8% and 9%, respectively), and vasopressor support (in 1% of patients in each group). Most ICANS events were grade 1–2. The most common signs and symptoms associated with ICANS included confusion, impaired attention, motor weakness, dysgraphia, and hypertonia. Neutropenia was reported in 17% of the 10 mg group and 16% of the 100 mg group, with grade 3 neutropenia noted in one patient from each group. Pyrexia occurred in 35% of the 10 mg group and 33% of the 100 mg group. Grade ≥ 3 AEs occurred in 59% and 64% of patients, respectively. Other AEs included constipation (27% and 25%, respectively), reduced appetite (29% and 44%, respectively), and anemia (26% and 25%, respectively). Patients reported improvement in dyspnea, chest pain, and cough after BsAb. Tarlatamab improved survival outcomes, and these results suggest that the BsAb is a much-needed addition to the therapeutic armamentarium in the current second-line therapies for patients with ES-SCLC [25].

Given that CRS was the most common AE that occurred in 53% of patients who received tarlatamab 10 mg in the DeLLphi-301 trial, it is essential to raise awareness for the management of these immune-related AEs. CRS mainly occurred after the first or second tarlatamab dose, and the most common CRS symptom, other than fever, was hypotension. Similarly, ICANS and associated neurologic events occurred in 10% of patients who were treated with tarlatamab 10 mg. Both CRS and ICANS are graded and managed according to the American Society for Transplantation and Cellular Therapy (ASTCT) consensus criteria [26–28].

Extended follow-up data from the DeLLphi-301 study were presented at the 2024 World Conference on Lung Cancer (WCLC). The results showed sustained anticancer activity and a manageable safety profile in patients with ES-SCLC who had previously been treated with platinum-based chemotherapy. Of the 100 patients treated with 10 mg of tarlatamab biweekly, the ORR was 40%, and the median duration of disease control was 6.9 months (95% CI, 5.4–8.6). The median OS for this group was 15.2 months after first-line platinum-based chemotherapy. No new safety signals were reported [29].

Currently, a phase-III DeLLphi-304 trial is underway, evaluating OS prolongation with tarlatamab and comparing it to the standard of care for previously treated ES-SCLC. Similarly, the phase-Ib DeLLphi-303 study is examining tarlatamab in combination with a PD-L1 inhibitor, plus carboplatin and etoposide, as first-line maintenance in ES-SCLC [30, 31].

### Tebentafusp-tebn

Tebentafusp-tebn is a bispecific TCE that binds to GP100 on tumor cells and CD3 on T-cells. It has been approved by the FDA for adult patients with HLA-A*02:01–positive unresectable or metastatic uveal melanoma based on the results of the IMCgp100-202 phase-III study [32]. In this trial, 252 patients were randomized to tebentafusp and 126 to control. Among controls, 103 received pembrolizumab, 16 received ipilimumab, and 7 received dacarbazine. The percentage of patients who achieved disease control in the tebentafusp and control arms was 46% and 27%, respectively. The mDOR was 9.9 months in the tebentafusp group and 9.7 months in the control group. The median duration of survival was 15.3 months in the tebentafusp group and 6.5 months in the control group, with ORRs of 9% and 5%, respectively. The PFS was 3.3 months in the tebentafusp group and 2.9 months in the control group. While 1-year OS rates were 73% versus 59%, the mOS was 21.7 months and 16 months, respectively.

AEs included rash (83%), pyrexia (76%), pruritus (69%), chills (47%), hypotension (38%), and erythema (23%). Grade 3–4 AEs occurred in 44% of the tebentafusp arm, with the most common AEs including rash (18%), pruritus (4%), pyrexia (4%), and hypotension (3%). In the tebentafusp group, 89% of patients developed CRS while 3% in the control arm. Overall, 12% of patients experienced grade 1 CRS, while 76% had grade 2 CRS. Only 1% of patients developed grade 3 CRS, and no cases of grade 4 or 5 CRS were reported. Notably, the trial protocol did not mandate prophylactic premedication for patients receiving tebentafusp. CRS management included supportive care (antipyretics, intravenous fluids, and glucocorticoids). Given that there is no standard of care for metastatic uveal melanoma, this novel immunotherapy represents a clinically meaningful new option. To improve patient outcomes [33, 34].

### Lifileucel

Lifileucel is an autologous TIL therapy, the first of its kind to be approved by the FDA for the treatment of unresectable or metastatic melanoma. TIL therapy involves the extraction of lymphocytes that directly infiltrate the tumor, followed by lymphodepletion chemotherapy (Cyclophosphamide 60 mg/kg/day × 2 days, Fludarabine 25 mg/m²/day × 5 days, High-dose IL-2: 600,000 IU/kg IV every 8 h, max 6 doses), cell infusion, and IL-2. This treatment is approved for patients who have previously been treated with a PD1 inhibitor or a serine/threonine kinase (BRAF) proto-oncogene, BRAF inhibitor, with or without a MEK inhibitor (if BRAF V600-positive) [35–37]. The TIL therapy was approved based on the results of the C-144-01 phase-II, single-arm, open-label, multicenter study [38]. Of the 189 patients initially recruited, 156 were treated with lifileucel. The primary reason for not receiving the infusion was disease progression. All patients had previously received treatment with an anti–PD-1 agent. Eighty-three patients (54.2%) had not responded to previous anti-PD-1–PD-1/PD-L1 therapy. The ORR was 31.5%, with a total of 8 CRs. The mDOR was not reached during the follow-up period. The mPFS was 4.1 months, and the mOS was 13.9 months. All patients experienced at least one TEAE. These included thrombocytopenia (82.7%), chills (75%), and anemia (62.2%). The most common grade≥3 AEs included thrombocytopenia (76.9%), anemia (50%), and febrile neutropenia (41.7%), with a safety profile largely consistent with the non-myeloablative lymphodepletion and IL-2 regimens. These AEs were reported as transient and manageable, consistent with the known toxicities of the lymphodepletion and high-dose IL-2 regimens. IRRs were infrequent (3.8%, grade 1–2). Other Immune-related adverse events were also infrequent. Capillary leak syndrome (attributed to IL-2) was reported in seven patients, while CRS and ICANS were observed in one patient. Intra-abdominal tumor hemorrhage and acute respiratory failure were also noted [38]. The findings from the C-144-01 clinical trial demonstrated that lifileucel has favorable clinical outcomes and presents as a novel therapy for advanced melanoma, with most AEs occurring within the first 2 weeks of therapy, mainly associated with IL-2 given after cell infusion [39].

The TIL construct is also being investigated in other disease settings. The IOV-COM-202 phase-II clinical trial explored the role of lifileucel in advanced NSCLC, metastatic melanoma, and metastatic head and neck squamous cell carcinomas (HNSCC). For NSCLC, the ORR was 21.4%, and the mDOR ranged from 1.1 to 26.2 months [36]. In metastatic melanoma, the ORR was 63.6%, with 22.7% of patients achieving a CR [40]. The most common Grade *≥* 3 AEs were thrombocytopenia (68.2%), neutropenia (50%), and anemia (45.5%). In patients with HNSCC, the ORR was 42.9% [41]. Lifileucel has also been investigated in cervical cancer in the phase-II trial C-145-04, where the ORR was 44% and the disease control rate (DCR) was 89% [42].

### Afamitresgene autoleucel

**Fig. 1:**
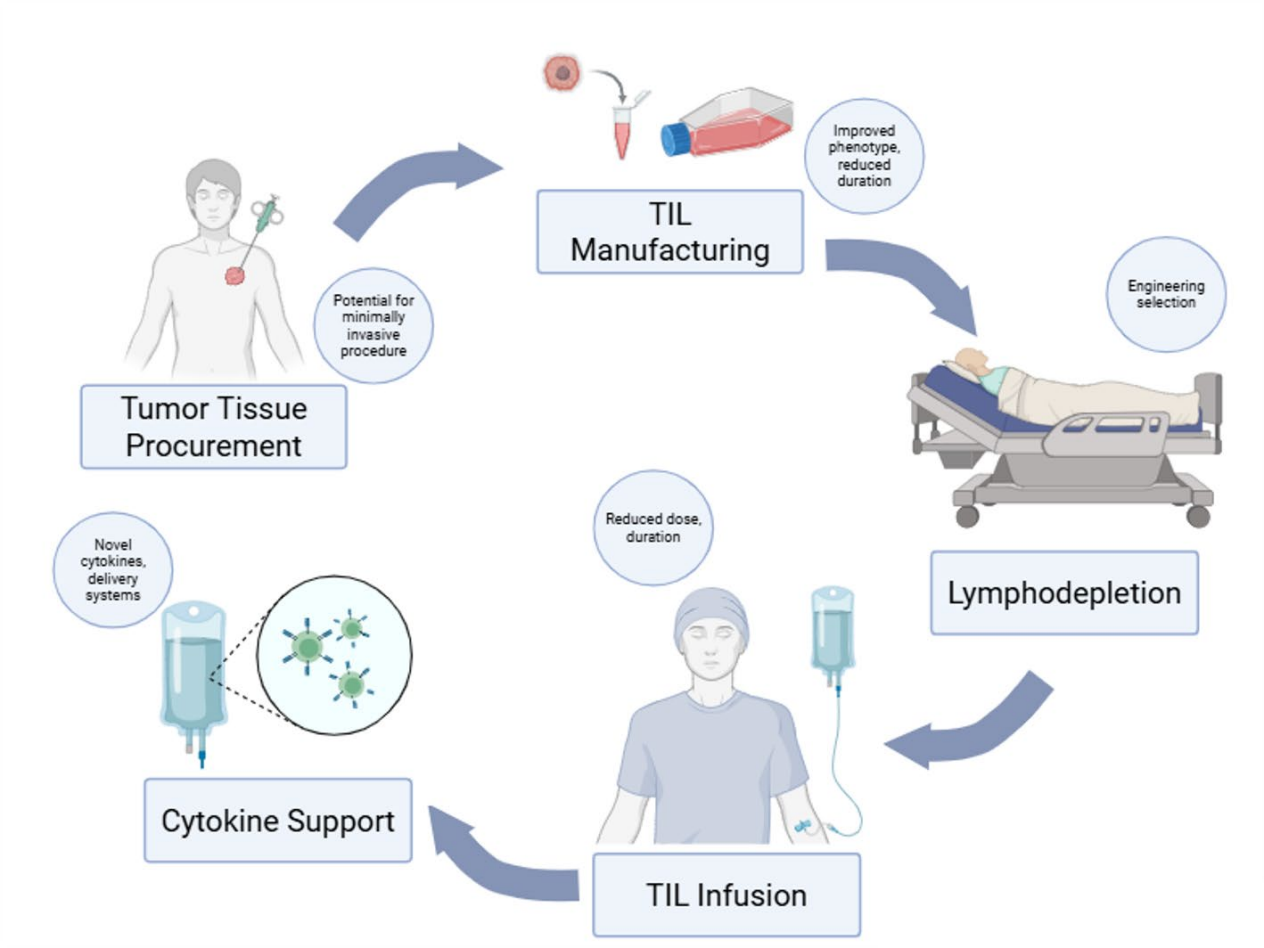
Depicts tumor-infiltrating lymphocyte constructs manufacturing

Afamitresgene autoleucel (afami-cel) is an autologous T cell therapy that targets melanoma-associated antigen A4 (MAGE-A4), a cancer/testis antigen that is expressed at different levels in a variety of solid tumors. As with other TCR-based platforms, recognition is MHC Class I-restricted, and afami-cel specifically targets an affinity-enhanced TCR directed against MAGE-A4 [43]. While commercially available CAR T-cells involve introducing an artificial receptor into immune effector cells to identify proteins on the surface of tumor cells [44, 45], TCR-engineered effector cells use naturally occurring or slightly modified TCRs to create T-cell–based adoptive therapy (Fig. 1). A TCR-based platform bears the ability to recognize tumor-specific epitopes presented by major histocompatibility complex (MHC) class I molecules on the surface of tumor cells. This strategy offers broader applicability, as there are significantly more tumor-specific sequences within cells presented by MHC than by tumor-specific proteins expressed on the cell surface. Although there aren’t many cell-surface antigens that antibodies can detect, estimates indicate that MHC molecules can present thousands of peptides, increasing the number of possible targets for TCR therapies. Even though CAR T-cells have historically targeted surface proteins, new CAR designs that can identify peptide-MHC complexes are becoming more prevalent, blurring the historical distinction between TCRs and CARs. Hence, unlike the CAR-based approach, these intracellular cancer targets can only be reached through TCR-based methods [46]. The distinction between TCR therapy and TIL therapy lies in their sources. TIL therapy utilizes lymphocytes residing within tumors, whereas TCR therapy involves isolating peripheral blood T-cells and genetically modifying them in vitro to express TCRs that target specific tumor antigens [36]. TIL and TCR therapy further differ in their practical requirements. TCR requires patient fitness for leukapheresis and is linked to HLA restrictions. Additionally, bridging therapy is often required during the production interval. TIL also requires accessible tumor tissue for manufacturing, a process that can have a lengthy production timeframe.

Afamitresgene autoleucel has been approved by the FDA for HLA-A*02-positive patients with unresectable or metastatic synovial sarcoma whose tumors express MAGE-A4, based on the results from the phase-II, open-label SPEARHEAD-1 clinical trial. Eligibility for the trial required tumor MAGE-A4 expression of ≥ 2 + staining intensity in ≥ 30% of tumor cells. It is approved for patients who have previously received chemotherapy and are HLA-A02:01P, -A02:02P, -A02:03P, or -A02:06P positive. In the study, 44 patients were treated with afamitresgene autoleucel, of which 2 patients achieved CRs. The ORR was 43.2%, the mDOR was 6 months, and the median PFS was 3.8 months. The median OS was 15.4 months, with an OS probability of 60% at 12 months and 40% at 24 months [47–50]. The ORR was 37% for synovial sarcoma and 25% for myxoid round-cell liposarcoma, respectively. All patients treated with afami-cel experienced at least one TEAE. All-grade AEs included CRS reported in 71% of patients, leukopenia (27%), pyrexia (23%), and neutropenia (23%). The majority of CRS events were grade 1 or 2, with one grade 3 event. 2 patients were administered corticosteroids, while 19 patients had tocilizumab for CRS management. Grade *≥* 3 AEs included lymphopenia in 96%, neutropenia in 85%, and leukopenia in 81% of patients [51]. Nausea, vomiting, fatigue, infections, pyrexia, constipation, dyspnea, abdominal pain, non-cardiac chest pain, decreased appetite, tachycardia, back pain, hypotension, diarrhea, and edema were also reported [48, 49]. In patients who experienced grade 1 CRS for ≥ 24 h or if the patients had comorbidities, tocilizumab was administered [51]. Patients received lymphodepleting chemotherapy with cyclophosphamide (600 mg/m^2^ daily for 3 days) and fludarabine (30 mg/m^2^ daily for 4 days) before afami-cel infusion. Afamitresgene autoleucel demonstrated clinically meaningful outcomes in patients with advanced sarcoma and lays the groundwork for novel TCR therapy to be further investigated across solid tumors.

### TCE therapeutic pipeline in solid tumors

Beyond these five FDA-approved agents, the therapeutic pipeline for solid tumors includes a number of other promising investigational therapies. Engineered TIL (eTIL) platforms have been developed to improve persistence and reduce the dependence on toxic, high-dose interleukin-2. One of the platforms was able to eliminate the need for IL-2 by developing membrane-bound IL-15 expression. This approach is promising, considering it can be regulated by acetazolamide (which acts as a switch to activate the cells’ anti-tumor function). An initial phase-I study using this novel approach has shown an ORR of 44.4% and a 24-week PFS rate of 75%. This was reported along with no cases of CRS, ICANS, and dose-limiting toxicities [52–54]. Gene-edited TIL approaches are also currently in development. These include platforms that employ CRISPR/Cas9 to knock out suppressive genes (SOCS1 and Regnase-1). These negatively regulate cytokine signaling by acting as intracellular checkpoints [55, 56].

HER-directed CAR-T cell therapies and bispecific antibodies are notable agents in the pipeline. Multiple distinct CAR-T cell therapies targeting HER2 are under investigation in various types of cancer, which include but are not limited to brain tumors, breast cancer, and advanced sarcomas [57–60]. Additionally, HER2-targeted bispecific antibodies are under evaluation, including those that target CD3 (Runimotamab), HER3 (Zenocutuzumab), and a “tribody” construct that engages CD16 [61–63].

Programs targeting prostate-specific membrane antigen (PSMA) include armored CAR T-cells designed to be TGFβ-insensitive, as well as PSMA/CD3 bispecific T-cell engagers (BiTE) such as pasotuxizumab and acapatamab [64–66].

For CAR-T, bispecific antibody, and BiTE platforms, numerous additional targets for solid tumors are the subject of ongoing research. These targets include claudin-18.2, EpCAM, ROR1, ENPP3, claudin-6, EGFR, GPC3, mesothelin (MSLN), MAGE-A4, MUC16, CEA, and B7-H6 [67–91]. These developments demonstrate the promise of targeted therapies in various cancers, encompassing a broad range of tumor-associated antigens.

When considered together, investigational programs share pre-treatment and toxicity mitigation strategies with the five approved agents. Management for CRS typically includes supportive care and, in some cases, administration of tocilizumab. Neurotoxicity is typically managed according to consensus guidance. Across these therapies, cytopenias, fever, and constitutional symptoms were reported. Furthermore, specific serious toxicities have been noted for certain classes of agents, such as Immune Effector Cell-associated Hemophagocytic Lymphohistiocytosis-like syndrome (IEC-HS) with some PSMA-targeted BiTEs. For TIL-based therapies, lymphodepleting chemotherapy and peri-infusional supportive care are standard. There are efforts to reduce manufacturing time for these, in addition to dependence on high doses of IL-2. These shared themes point towards the importance of a systematic approach and management algorithms for better comparison across the platforms.

### Potential strategies to optimize TCEs in solid tumors

Several challenges remain for the broad implementation of T-cell redirecting therapies in solid tumors. These can be attributed to cytokine release, manufacturing time, patient selection, and HLA-restricted applicability. Strategies can be learned and devised to address these barriers. For instance, prophylactic use of glucocorticoids and a step dosing approach for tarlatamab can be used to attenuate CRS. High prevalence of CRS was observed in the tebentafusp trial, as prophylactic measures were not mandatory before the administration of the agent. The cases, however, were managed conservatively. TIL therapy with lifileucel highlighted the impact of logistic hurdles. These hurdles, which included the period from tumor resection to infusion, led to disease progression in a few patients, resulting in them being ineligible to participate. Finally, TCR therapies like afamitresgene autoleucel highlight the tradeoff between precise targeting and the limitations of HLA restriction, as the therapy is restricted to patients with specific HLA-A*02 alleles.

Beyond these clinical insights, design-related issues remain pertinent. There have been reports of anti-drug antibody formation with some therapies, as seen with tebentafusp. While they developed in 29% of the patients, it did not have any apparent impact on the overall survival. HLA restriction restricts the eligible candidates’ participation in TCR therapies, as observed with afami-cel. The efficacy of these agents is severely undermined by an immunosuppressive tumor microenvironment.

While CAR-T therapies have revolutionized the treatment paradigms for hematologic malignancies, their efficacy in solid tumors has been limited. These can be linked to the tumor-induced immunosuppressive tumor microenvironment and paucity of tumor-specific surface proteins.

To improve safety and tolerability, new strategies are being explored. These include efforts like the development of alternative IL-2 analogs in order to mitigate the toxicities associated with high-dose IL-2 used in TIL therapy.

Lessons from hematologic malignancies also inform progress in solid tumors. The importance of lymphodepletion for cellular therapy expansion can be deduced from the fact that it is a required component for the administration of lifileucel and afami-cel.

For the success of these therapies, infrastructure is another important consideration. Since TILS (lifileucel) and TCR-based therapies (afami-cel) require lymphodepleting chemotherapy. These cannot be performed in an outpatient setting, resulting in the need for inpatient care. Other agents, such as tebentafusp, also require hospitalization during the initial phase. The patients are monitored overnight for the first three weeks of dose escalation. This underscores that resource-intensive supportive care is a shared challenge across these advanced modalities.

Another key strategy to optimize these therapies involves combining them with other treatments to overcome resistance and enhance efficacy. In the DeLLphi-303 trial, a combination of PD-L1 inhibitor and tarlatamab is being examined as first-line therapy. Similarly, the value of combining TIL therapy with immune checkpoint inhibitors is a major area of investigation. Lifileucel in combination with pembrolizumab is being evaluated for patients with melanoma. These approaches offer promising avenues for leveraging the distinct mechanisms of action of these drugs to enhance their efficacy against cancer.

Another promising strategy to enhance tumor specificity is the targeting of aberrant glycoforms of tumor-associated antigens. The glycosylation patterns are frequently altered in malignancies. These glycans, specific to cancer, serve as unique targets for CARs or BsAbs. This also offers the potential to minimize off-tumor toxicity on normal tissue (the same core protein but a different glycosylation pattern) [90].

### Future directions

T-cell immunotherapy is a novel treatment method for solid tumors. BsAbs, autologous TIL therapy, and autologous TCR therapy have the potential to improve patient outcomes and prolong survival. These therapies can serve as adjuncts to current standards of care and present plausible alternatives. Given the limited data on FDA-approved immunotherapies for solid tumors, there is a need to expand clinical research on these treatments and increase access for eligible candidates. However, there are risks of AEs, including infections, CRS, hematologic abnormalities, and IRR. Overall, these therapies have the potential to revolutionize the management of advanced solid tumors.

While the current landscape is dominated by T-cell-engaging therapies, there are efforts to overcome the limitations (including trafficking issues and on-target/off-tumor toxicity) for conventional CAR-T cells. Among these, Chimeric Antigen Receptor Natural Killer T (CAR-NKT) have attracted attention, though currently in early phase/pre-clinical development. Their inherent antitumor properties allow them to mitigate components of immunosuppression, specifically through targeting CD1d-expressing tumor-associated macrophages and MDSCs within the TME. Recent data (preclinical studies) have highlighted superior antitumor efficacy and a favorable safety profile for CAR-NKT cells compared to CAR-T cells in solid tumor models [91]. Furthermore, the development of ‘off-the-shelf’ allogeneic CAR-NK and CAR-NKT therapies represents a critical area of investigation that could democratize access to these complex treatments and reduce manufacturing timelines.

## Data Availability

Data sharing is not applicable to this article as no datasets were generated or analyzed during the current study.

## Abbreviations

AEs: Adverse events
ASTCT: American society for transplantation and cellular therapy
BRAF: serine/threonine kinase
BsAbs: Bispecific antibodies
CAR: Chimeric antigen receptor
CR: Complete responses
CRS: Cytokine release syndrome
DCR: Disease control rate
DIPG: Diffuse intrinsic pontine glioma
EGFR: Epidermal growth factor receptor
ELCC: European lung cancer congress
ES-SCLC: Extensive-stage small cell lung cancer
eTIL: Engineered tumor-derived autologous T-cell immunotherapy
FDA: Food and drug administration
GBM: Glioblastoma multiforme
HCC: Hepatocellular carcinoma
ICANS: Immune effector cell–associated neurotoxicity syndrome
IRR: Infusion-related reactions
mDOR: Median duration of response
MET: Mesenchymal epithelial transition
MHC: Major histocompatibility complex
NK: Natural killer
NSCLC: Non–small cell lung cancer
ORR: Overall response rate
OS: Overall survival
PFS: Progression-free survival
TAMs: Tumor-associated macrophages
TCE: T-cell–engaging
TCR: T-cell receptor
TILs: Tumor-infiltrating lymphocytes
TME: Tumor microenvironment
TNBC: Triple-negative breast cancer
Tregs: Regulatory T-cells
WCLC: World conference on lung cancer
